# Prevalence of Neoplastic Diseases in Pet Birds Referred for Surgical Procedures

**DOI:** 10.1155/2016/4096801

**Published:** 2016-02-15

**Authors:** Patrícia F. Castro, Denise T. Fantoni, Bruna C. Miranda, Julia M. Matera

**Affiliations:** Department of Surgery, School of Veterinary Medicine and Animal Science, University of São Paulo, Avenida Prof. Orlando Marques de Paiva 87, Cidade Universitária, 05508-270 São Paulo, SP, Brazil

## Abstract

Neoplastic disease is common in pet birds, particularly in psittacines, and treatment should be primarily aimed at tumor eradication. Nineteen cases of pet birds submitted to diagnostic and/or therapeutic surgical procedures due to neoplastic disease characterized by the presence of visible masses were retrospectively analyzed; affected species, types of neoplasms and respective locations, and outcomes of surgical procedures were determined. All birds undergoing surgery belonged to the order Psittaciformes; the Blue-fronted parrot (*Amazona aestiva*) was the prevalent species. Lipoma was the most frequent neoplasm in the sample studied. Most neoplasms affected the integumentary system, particularly the pericloacal area. Tumor resection was the most common surgical procedure performed, with high resolution and low recurrence rates.

## 1. Introduction

Pet birds suffer from a wide variety of neoplastic diseases [1]. Growing understanding of avian medicine is increasingly turning neoplastic diseases into more than just a* postmortem* diagnosis; however, related scientific literature, particularly therapeutic data, remains scarce [2, 3] or is limited to case reports [4, 5].

Neoplastic diseases are common among pet birds, particularly psittacines [6]; lipomas, lymphomas, and fibrosarcomas are among the most common neoplasms seen in birds in the genus* Amazona* [7]. Therapeutic strategies should be aimed at tumor eradication and may involve several modalities, employed as either combined or staged treatments [8]. Whenever feasible, surgical resection of neoplastic masses is the treatment of choice [1, 9]; however, radiotherapy, photodynamic therapy, cryotherapy, and chemotherapy may also be used [8].

This study set out to determine which bird species, among those presented to our referral veterinary center, are most frequently affected with neoplastic diseases characterized by the presence of visible masses, as well as neoplasm types and locations. Outcomes of different surgical procedures performed in affected pet birds were also analyzed.

## 2. Materials and Methods

**Figure 1:**
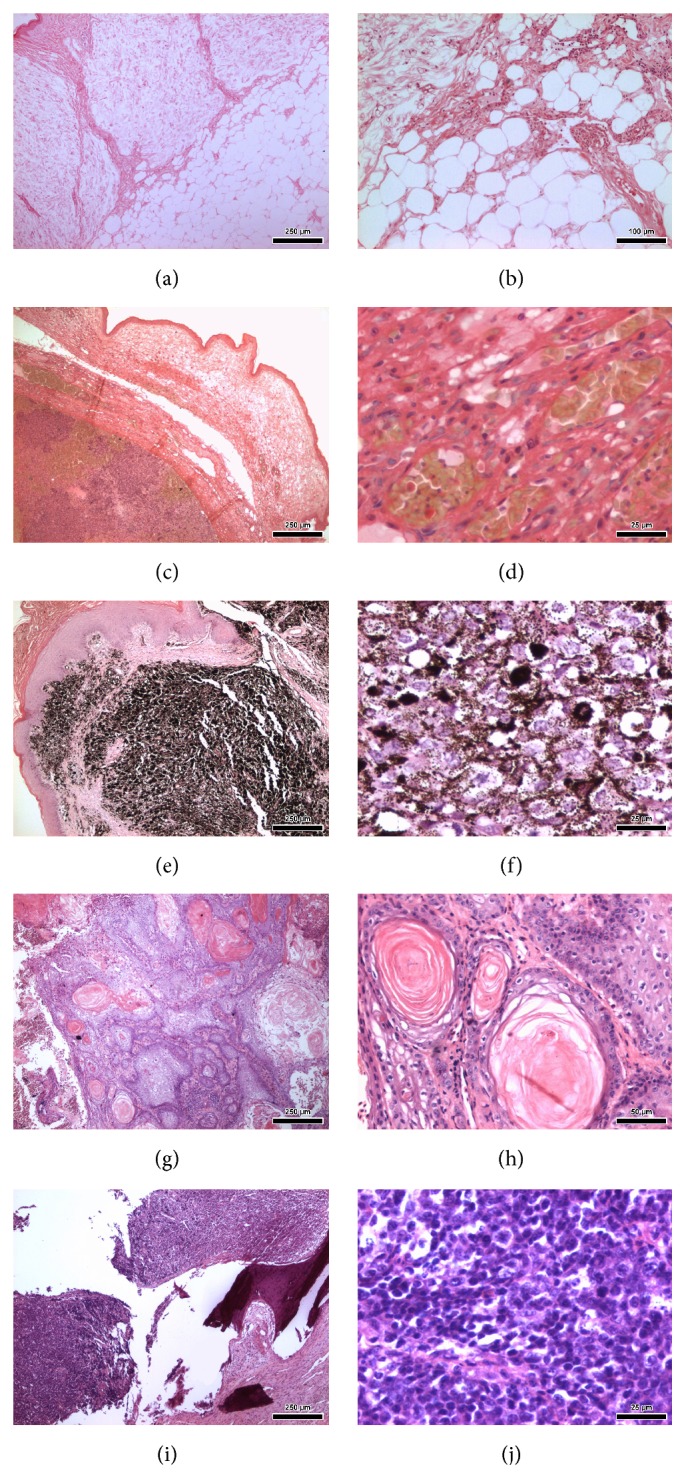
Histological photomicrographs of avian neoplasms—Hematoxylin and Eosin stain (H&E). (a) and (b) Case number 1: pericloacal lipoma (*A. aestiva*). (c) and (d) Case number 2: well-differentiated hemangiosarcoma in pelvic limb (*A. aestiva*). (e) and (f) Case number 3: mandibular melanoma (*Ara ararauna*). (g) and (h) Case number 11: oral squamous cell carcinoma (*Diopsittaca nobilis*). (i) and (j) Case number 15: distal tibiotarsal lymphoma (*Amazona* sp.).

Data regarding bird species, type of neoplasm (according to histological diagnosis; Figure 1) and respective location, type of surgical procedure, and short- (one week) and long-term progression were collected from pet birds submitted to diagnostic and/or therapeutic surgical procedures at the Small Animal Surgery Department of the Veterinary Hospital of the School of Veterinary Medicine and Animal Science, University of São Paulo (FMVZ/USP), over an eight-year period.

**Figure 2:**
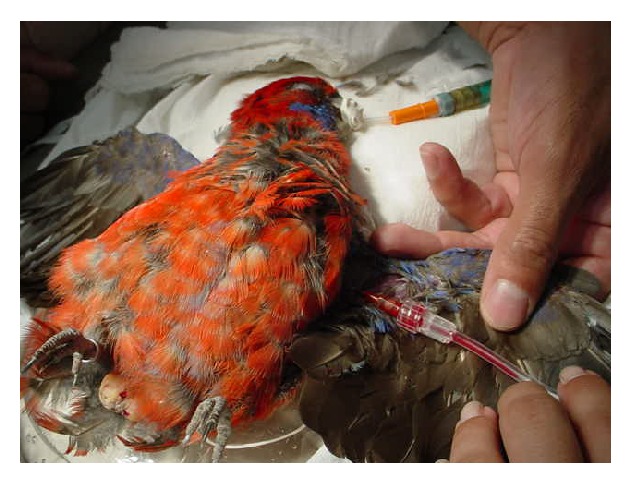
Case number 16: eastern rosella (*Platycercus eximius*) undergoing preoperative procedures. Note the endotracheal tube (adapted urinary catheter) in place and the catheterization of the brachial vein for intraoperative fluid therapy.

The anesthetic protocol included preanesthetic medication with intramuscular diazepam (Compaz 10 mg injectable; Cristália, Itapira, SP) and ketamine (Dopalen injectable; Agribrands do Brasil, Paulínia, SP) (pectoral muscle; 1 mg/kg and 10 mg/kg, resp.) and induction with sevoflurane (Sevocris, Cristália; Itapira, SP) in 100% oxygen delivered via facial mask in a closed nonrebreathing circuit; an appropriate sized endotracheal tube was then passed for anesthetic maintenance. Birds received fluid therapy (lactated ringer solution; 10 mL/kg/h) via a catheter inserted into the brachial vein (Figure 2). Intramuscular flunixin-meglumine (Banamine injectable 10 mg; Schering-Plough, Rio de Janeiro, RJ) (5 mg/kg) was given for immediate postoperative pain management.

Anesthetized birds were further prepared for surgery. Following feather plucking and removal from the surgical field, birds were placed in the required position for the procedure at hand and skin antisepsis performed with 70% alcohol and povidone iodine, taking care to avoid excessive wetting and potential body temperature loss. Intramuscular enrofloxacin (Baytril 5% injectable; Bayer, São Paulo, SP) (pectoral muscle; 15 mg/kg) was also given. Surgical procedures were performed according to recommendations given in literature regarding minimal surgical trauma and bleeding [9–11]. Skin closure was achieved with 4-0/5-0 nylon, 4-0 poliglecaprone, or 4-0 polyglactin 910, in a simple interrupted pattern.

Short-term progression was graded* excellent* (complete resolution),* satisfactory* (resolution not achieved but diagnosis confirmed),* unsatisfactory* (deterioration of patient's clinical condition), or* death* (death within 24 hours of surgery). Long-term progression was characterized as* no* (no recurrence along the experimental period),* yes* (recurrence along the experimental period),* death*,* lost to follow-up*, or* euthanasia*.

## 3. Results

Nineteen diagnostic and/or therapeutic surgical procedures were performed in pet birds during the eight-year experimental period, which then led to patient identification; one bird was operated on three times, each accounting for one procedure in the sample. All birds belonged to the order Psittaciformes, with birds in the genus* Amazona* accounting for 84.21% (16/19) of cases. The Blue-fronted parrot (*Amazona aestiva*) was the most prevalent species (36.84%, 7/19). Malignant and benign tumors accounted for 31.57% (6/19) and 68.42% (16/19) of lesions, respectively. All benign tumors in this sample were lipomas and were more commonly diagnosed in birds in the genus* Amazona* (92.30%, 12/13) (Table 1).

**Figure 3:**
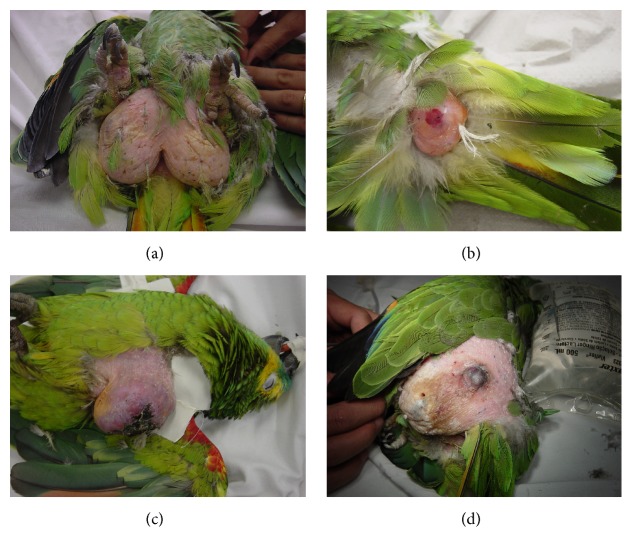
Neoplasms affecting the integumentary system of birds in the genus* Amazona.* (a) Pericloacal lipoma (*A. aestiva*). (b) Lipoma affecting the dorsal area near the tail (*Amazona* sp.). (c) Cutaneous lymphoma (*A. aestiva*). (d) Well-differentiated hemangiosarcoma affecting the pelvic limb (*A. aestiva*).

Most neoplasms affected the integumentary system. Neoplastic lesions were limited to the pericloacal area in 42.10% of cases (8/19; Figure 3(a)) and extended to the abdominal area or the pelvic limb in three (15.78%, 3/19) and one case (5.26%, 1/19), respectively. With the exception of one case (liposarcoma), integumentary pericloacal lesions were diagnosed as lipomas. Other sites affected by neoplasms in this study were the abdominal area (lipoma; 5.26%, 1/19), the dorsal area near the tail (lipoma; 5.26%, 1/19; Figure 3(b)), the chest (lymphoma; 5.26%, 1/19; Figure 3(c)), the distal tibiotarsal area (lymphoma; 5.26%, 1/19), the oral cavity (well-differentiated squamous cell carcinoma; 5.26%, 1/19), the pelvic limb (well-differentiated cutaneous hemangiosarcoma; 5.26%, 1/19; Figure 3(d)), and the mandible (melanoma; 5.26%, 1/19).

**Figure 4:**
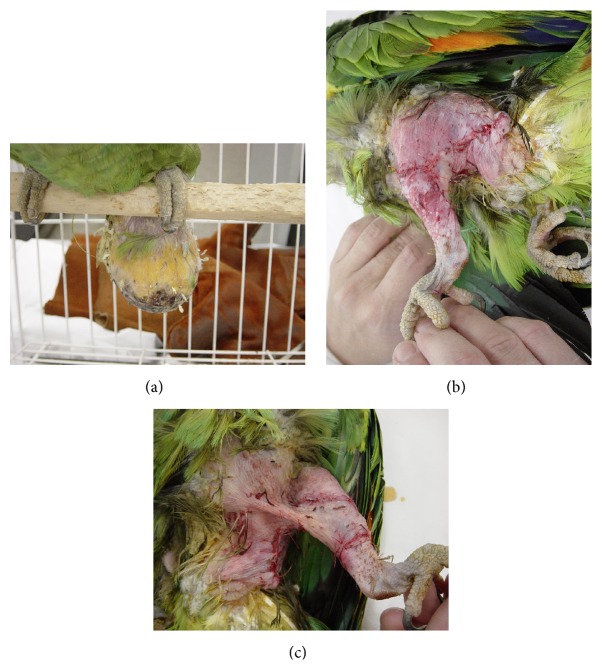
Case number 13: lipoma affecting the pelvic limb and pericloacal area (*Amazona* sp.). (a) Caudal, ulcerated, pendulum-like neoplasm. ((b) and (c)) Immediate postoperative appearance: skin closure on the lateral (b) and medial (c) aspects of the pelvic limb, extending to the pericloacal area (simple interrupted sutures, 4-0 polyglactin 910).

Short-term postoperative data were lacking in two cases; therefore, those were excluded from the analysis. Analysis of the remaining cases revealed the following surgical interventions: resection, resection and cryotherapy, incisional biopsy, and collection of samples for histopathological and/or culture and sensitivity testing. No birds showed deterioration of clinical condition (*unsatisfactory*) or died within 24 hours of surgery (*death*). Resection (Figure 4) was the most common surgical procedure performed (82.35%, 14/17), with excellent short-term outcomes in 100% of cases (14/14; complete resolution). These included all lipomas (12/14), one well-differentiated hemangiosarcoma (1/14), and one liposarcoma (1/14), with a 14.28% recurrence rate over the long-term (2 lipomas out of 14 tumors resected; Table 2).

## 4. Discussion

In this study, all birds affected with neoplastic disease belonged to the order Psittaciformes; the Blue-fronted parrot (*Amazona aestiva*) was the prevalent species. A study conducted at Northwest ZooPath specialty diagnostic service (Monroe, WA) reported higher prevalence of tumors in Anseriformes; Psittaciformes was the fifth most prevalent order, with cockatiels (*Nymphicus hollandicus*) and parrots (*Amazona *sp.) accounting for the first and second most frequently affected species [12]. Prevalence and popularity of different birds around the world [13] may explain these discrepancies; birds in the genus* Amazona* are likely the best known among New World psittacines and 27 members of this genus can be found throughout the Caribbean and Central and South America [7]. Results of this study are further supported by scientific data suggesting higher prevalences of neoplasia in Psittaciformes [8, 9, 14, 15], which possibly reflects the global popularity of psittacines as pets birds [16].

As reported elsewhere [17, 18], lipomas were the most common neoplasms in this study and tended to affect primarily birds in the genus* Amazona* [7]. The fact that most of these birds were obese suggests that high energy diets may play a role in the etiology of lipomas, along with genetic predisposition [1–3]. Lipomas have often been reported in budgerigars [3] and cockatoos [6, 19, 20]. Population composition clearly influenced the outcomes of this study. Native parrots are ubiquitous pet birds in Brazil [21]; therefore birds in the genus* Amazona* accounted for most cases of neoplasia in this sample. Also, higher surgical morbidity and mortality in small sized birds [22] translates into lower numbers of such patients (e.g., budgerigars) being submitted to surgical procedures given the high risk of death and related owner concerns.

Most neoplasms in this sample affected the integumentary system. A literature survey of neoplasia in pet birds, including cases diagnosed at a Veterinary Medical Teaching Hospital (University of California, Davis) over a 10-year period, revealed that tumors arising in the integument (31.7%) were more common than from other organ systems [6]. Lipomas were more frequently reported in the sternum, abdomen, and inner face of the thigh [8]; in contrast, in our study the pericloacal area was the most commonly affected site.

Resection was the most common surgical procedure [1, 9] performed (14/17), with excellent short-term outcomes (i.e., complete resolution) in 100% of cases. The long-term recurrence rate following surgical resection was 14.28% (2/14) and reflected disease progression in the same patient (cases numbers 4, 5, and 6; Table 2), operated on three times over the course of the eight-year experimental period for resection of pericloacal lipomas. Lipomas tend not to have well-defined margins, with tumor blood vessels often infiltrating the surrounding adipose tissue; resection is therefore more difficult and recurrence more common in such cases [2, 17]. In case 17, the neoplasm (lipoma) was treated with a combination of surgical resection and cryotherapy and did not recur. Aspiration cytology findings in this case suggested liposarcoma, but tumor location close to the cloaca precluded resection with sufficiently wide surgical margins. Hence a combined procedure was performed. Cryotherapy is indicated for treatment of tumors located around the oral cavity and nares, or as an ancillary technique in cases involving resection of wide-based or malignant tumors such as fibrosarcoma [8].

Short-term outcomes of incisional biopsies (2/17) and collection of samples for histopathology or culture/sensitivity testing (1/17) were satisfactory (i.e., resolution not achieved but diagnosis confirmed) in 100% of cases in this study. In the long-term, all (100%) of these cases progressed to death and/or were euthanized; this was not surprising given the diagnoses of melanoma, lymphoma (cases numbers 3 and 15, resp.; Table 2), and advanced oral squamous cell carcinoma (case number 11; Table 2), all malignant tumors, with limited possibilities of quality of life improvement via palliative and/or specific antineoplastic treatment. Different from birds suffering from benign conditions (e.g., lipomas), in the aforementioned cases, surgical procedures were intended for diagnosis rather than disease eradication. Still surgical interventions provided support for therapeutic decisions aimed at quality of life improvement and are therefore indicated in cases of neoplastic disease with a poor prognosis in birds.

Deeper understanding of neoplastic diseases amenable to surgical treatment in pet birds, as well as data on affected species, tumor prevalence, preferential location of neoplasms, and potential outcomes of related surgical interventions, constitutes relevant information for clinicians specializing in avian medicine. Also important is the fact that such knowledge may serve as a basis for future studies in avian oncology.

## 5. Conclusion

All birds operated on due to neoplastic disease in this study belonged to the order Psittaciformes, with a higher prevalence of birds in the genus* Amazona*. Lipoma was the most prevalent neoplasm and the pericloacal area the most commonly affected site. Lipomas responded well to surgical resection, with high complete resolution and low recurrence rates. Diagnostic procedures such as incisional biopsy provided support for therapeutic planning in cases with a poor prognosis.

## Figures and Tables

**Table 1:** Numerical (*N*) and percentage (%) distribution of soft tissue neoplasms in different species of Psittaciformes submitted to surgical interventions at the FMVZ/USP Veterinary Hospital between 2000 and 2008, São Paulo, Brazil.

Common name *Scientific name*	Lipoma		Lymphoma		Liposarcoma		Hemangiosarcoma		Squamous cell carcinoma		Melanoma		Total	
	*N*	%	*N*	%	*N*	%	*N*	%	*N*	%	*N*	%	*N*	%
Blue-fronted parrot *Amazona aestiva*	4	21.05	1	5.26	1	5.26	1	5.26	0	0.00	0	0.00	**7**	**36.84**
Orange-winged parrot *Amazona amazonica*	6	31.57	0	0.00	0	0.00	0	0.00	0	0.00	0	0.00	**6**	**31.57**
Parrot *Amazona* sp.	2	10.52	1	5.26	0	0.00	0	0.00	0	0.00	0	0.00	**3**	**15.78**
Blue-and-yellow macaw *Ara ararauna*	0	0.00	0	0.00	0	0.00	0	0.00	0	0.00	1	5.26	**1**	**5.26**
Red-shouldered macaw *Diopsittaca nobilis*	0	0.00	0	0.00	0	0.00	0	0.00	1	5.26	0	0.00	**1**	**5.26**
Eastern rosella *Platycercus eximius*	1	5.26	0	0.00	0	0.00	0	0.00	0	0.00	0	0.00	**1**	**5.26**
Total	**13**	**68.42**	**2**	**10.52**	**1**	**5.26**	**1**	**5.26**	**1**	**5.26**	**1**	**5.26**	**19**	**100.00**

**Table 2:** Distribution of 17 cases of neoplastic disease operated on between 2000 and 2008 at FMVZ/USP, São Paulo, Brazil, according to surgical outcomes.

Case number	Common name (*Scientific name*)	Soft tissue neoplasm	Surgical procedure	Short-term progression		Long-term progression				
				E	S	No	Yes	D	NI	Eut
1	Blue-fronted parrot (*Amazona aestiva)*	Pericloacal lipoma	Resection	x		x				
2	Blue-fronted parrot (*Amazona aestiva*)	Well-differentiated hemangiosarcoma in pelvic limb	Resection	x		x				
3	Blue-and-yellow macaw (*Ara ararauna*)	Mandibular melanoma	Incisional biopsy		x			x		
4	Orange-winged parrot (*Amazona amazonica*)	Pericloacal lipoma	Resection	x			x			
5	Orange-winged parrot (*Amazona amazonica*)	Pericloacal lipoma	Resection	x			x			
6	Orange-winged parrot (*Amazona amazonica*)	Pericloacal lipoma	Resection	x		x				
7	Blue-fronted parrot (*Amazona aestiva*)	Pericloacal liposarcoma	Resection	x		x				
8	Orange-winged parrot (*Amazona amazonica*)	Abdominal and pericloacal lipoma	Resection	x		x				
9	Orange-winged parrot (*Amazona amazonica*)	Pericloacal lipoma	Resection	x		x				
10	Blue-fronted parrot (*Amazona aestiva*)	Abdominal ventral lipoma	Resection	x		x				
11	Red-shouldered macaw (*Diopsittaca nobilis*)	Oral squamous cell carcinoma	SC (HP, CST)		x					x
12	Blue-fronted parrot (*Amazona aestiva*)	Abdominal and pericloacal lipoma	Resection	x		x				
13	Parrot (*Amazona* sp.)	Pelvic limb and pericloacal lipoma	Resection	x		x				
14	Blue-fronted parrot (*Amazona aestiva*)	Pericloacal lipoma	Resection	x		x				
15	Parrot (*Amazona* sp.)	Distal tibiotarsal lymphoma	Incisional biopsy		x			x		
16	Eastern rosella (*Platycercus eximius*)	Pericloacal lipoma	Resection	x					x	
17	Parrot (*Amazona* sp.)	Lipoma on dorsum near the tail	Resection and cryotherapy	x		x				
Total				**14**	**3**	**11**	**2**	**2**	**1**	**1**

*Surgical procedure*: SC: sample collection (HP: histopathology; CST: culture and sensitivity testing); *short-term progression*: E: excellent (complete resolution); S: satisfactory (resolution not achieved but diagnosis confirmed); *long-term progression*: no: no recurrence along the experimental period; yes: recurrence along the experimental period; D: death; NI: information not available; Eut: euthanasia.
